# Cytoreductive surgery and perioperative intraperitoneal chemotherapy for peritoneal carcinomatosis in the elderly

**DOI:** 10.1186/s12957-015-0682-7

**Published:** 2015-08-28

**Authors:** Yeqian Huang, Nayef A. Alzahrani, Saleh E. Alzahrani, Jing Zhao, Winston Liauw, David L. Morris

**Affiliations:** St George Clinical School, University of New South Wales, St George Hospital, New South Wales, Australia; Department of Surgery, University of New South Wales, St George Hospital, New South Wales, Australia; College of Medicine, Imam Muhammad ibn Saud Islamic University, Riyadh, Saudi Arabia; Department of Medical Oncology, St George Hospital, Sydney, New South Wales Australia; Hepatobiliary and Surgical Oncology Unit, Department of Surgery, St George Hospital, University of New South Wales, Level 3 Pitney Building, Gray Street, Kogarah, Sydney, NSW 2217 Australia

**Keywords:** Cytoreductive surgery, Peritonectomy, Peritoneal carcinomatosis, Elderly

## Abstract

**Background:**

Peritoneal carcinomatosis is life-threatening without cytoreductive surgery (CRS) and perioperative intraperitoneal chemotherapy (PIC). Only a few studies in the literature addressed the relationship between age and outcomes of peritonectomy. This study was designed to review the clinical outcomes in elderly patients who underwent CRS and PIC.

**Methods:**

This is a retrospective study of prospectively collected data of 611 consecutive patients with peritoneal carcinomatosis who underwent CRS and PIC by the same surgical team at St George Hospital in Sydney, Australia, between January 1996 and December 2013. Patients were divided into two groups; group 1 (<65 years old, *n* = 487) and group 2 (≥ 65 years old, *n* = 124). Subgroup analysis was performed in patients who were ≥75 years old (*n* = 20). A significant difference was defined as *p < 0.05*.

**Results:**

There was no significant statistical difference in terms of mean total hospital stay, intensive care unit stay, high dependency unit stay and complication rates. Postoperative mortality was 2 and 3 % in groups 1 and 2, respectively. Overall survival did not reach a statistical significance between the two groups. In subgroup analysis, patients showed similar morbidity results to patients who were <65 years old.

**Conclusions:**

CRS and PIC can be safely done in the elderly. Age alone should not be the single exclusion criterion but rather taken into consideration along with other factors to determine the suitability of elderly patients.

## Background

Peritoneal carcinomatosis is considered as a fatal condition without surgical and intraperitoneal chemotherapy interventions [1, 2]. Common malignancies involved include appendiceal cancer, colorectal cancer (CRC), mesothelioma, gastric cancer and ovarian cancer. Peritoneal carcinomatosis is currently treated with a combination of cytoreductive surgery (CRS) and perioperative intraperitoneal chemotherapy (PIC) by using the coliseum technique developed by Sugarbaker in the 1990s [3, 4]. PIC includes hyperthermic intraperitoneal chemotherapy (HIPEC) after CRS, and also, normothermic intraperitoneal chemotherapy is used by some units in the postoperative period (EPIC) [5]. A recent systematic review done by Chua et al. has shown an optimal morbidity rate ranging from 12 to 52 % and a mortality rate ranging 0.9 and 5.8 % [6]. A considerable number of patients with peritoneal carcinomatosis are aged 60 or older and are more commonly associated with comorbidities due to their age [7, 8]. Thus, whether the elderly can tolerate such an invasive surgery presents a challenge for healthcare professionals. A few studies have previously investigated the relationship between age and the outcome of peritonectomy [7–9]. However, these studies were limited by their small sample size.

The aim of this study was to review the short-term clinical outcomes and long-term survival in elderly patients who underwent CRS and PIC.

## Methods

### Settings

This is a retrospective study of prospectively collected data of 611 consecutive patients with peritoneal carcinomatosis who underwent CRS and PIC by the same surgical team at St George Hospital in Sydney, Australia, between January 1996 and December 2013. All the clinical and treatment-related data were collected and entered into a computerised database in order to evaluate the perioperative outcomes of patients with peritoneal surface malignancy. A signed informed consent was obtained from all patients. This research was ethically approved by the Human Research Ethics Committee at South Eastern Sydney Local Health District, NSW, Australia.

### Patients

All patients were managed by a standard treatment protocol combining CRS and PIC. Suitability to undergo CRS and PIC was evaluated during a regular weekly meeting attended by a multidisciplinary team including surgical oncologists, medical oncologists, radiologists, cancer care nurses and research staff. Selection criteria in our centre include (1) proven peritoneal disease; (2) no extra-peritoneal evidence of disease on preoperative evaluation including abdominal and chest computed tomography (CT), CT-angiogram of the liver and positron emission tomography (PET) scan; (3) able to give adequate informed consent; (4) performance status (World Health Organization performance status ≤2); (5) no severe co-existent visceral disease; (6) adequate renal, hepatic and haematological reserve; (7) acceptable intra-operative Peritoneal Cancer Index (PCI ≤ 15 or maximum of four liver metastases and PCI ≤ 10 for patients with CRC; PCI < 10 for patients with gastric cancer; no PCI limit for appendiceal cancer, peritoneal mesothelioma, pseudomyxoma peritonei (PMP) and ovarian cancer); and (8) appropriate histological subtype for given tumour type (exclude those with unfavourable subtypes with a very high PCI, e.g. mucinous adenocarcinomas with signet ring differentiation and sarcomatoid-variant peritoneal mesothelioma).

In this study, 611 patients were divided into two groups. Group 1 included patients who were less than 65 years old at the time of surgery (*n* = 487). Group 2 included patients who were 65 years of age or older at the time of surgery (*n* = 124). A subgroup analysis was performed for patients who are 75 years of age or older at the time of surgery.

### Preoperative management

All patients underwent standard preoperative investigations which included physical examination; tumour markers; double contrast-enhanced CT scans of the chest, abdomen and pelvis; and CT-angiogram of the liver with PMP and CRC. PET scan was performed in patients with appendiceal adenocarcinoma, CRC and mesothelioma.

### CRS

An initial assessment of the volume and extent of disease was recorded using the PCI, as described by Jacquet and Sugarbaker [10]. This assessment combines thickness of lesion size (LS) (LS 0: no macroscopic tumour; LS 1: tumour <0.5 cm; LS 2: tumour 0.5–5 cm; and LS3: tumour >5 cm) with tumour distribution (abdominopelvic region 0–12) to quantify the extent of disease as a numerical score (PCI 0–39). CRS was performed using Sugarbaker’s technique [11]. The aim of CRS was to remove all macroscopic intraperitoneal and visceral tumour deposits.

All sites and volumes of residual disease following CRS were recorded prospectively using the completeness of cytoreductive (CC) score (CC0—no macroscopic residual cancer remained; CC1—no nodule >2.5 mm in diameter remained; CC2—nodules between 2.5 mm and 2.5 cm in diameter remained; CC3—nodules >2.5 cm in diameter remained) [10]. Perioperative complications in all patients were graded I to IV with increasing severity based on the Clavien-Dindo classification of surgical complications (grade I: no treatment; grade II: medications only; grade III: surgical, endoscopic or radiological intervention; grade IV: life-threatening complications requiring intensive care unit (ICU) admission) [12].

### HIPEC

After CRS, but prior to intestinal anastomosis or repair of seromuscular tears, HIPEC was performed by installation of a heated chemoperfusate into the abdomen using the coliseum technique at approximately 42 °C for 30 or 90 min during CRS, depending on tumour types. For PMP, mitomycin C (12.5 mg/m^2^ over 90 mins) was used. For peritoneal mesothelioma, cisplatin (100 mg/m^2^) and mitomycin (12.5 mg/m^2^) in 1000 ml normal saline were given over 90 min. For appendiceal adenocarcinoma and CRC, oxaliplatin 350 mg/m^2^ in 500 ml of 5 % dextrose was given over 30 mins.

### EPIC

The criteria for EPIC include absence of leakage of the intraperitoneal chemotherapy system, absence of major organ failure and the ability of the patient to tolerate increased intra-abdominal fluid volume and intra-abdominal pressure with adequate urine output. EPIC was withheld if patients were haemodynamically unstable or experienced early perioperative complications.

The sump drains were clamped during the EPIC infusion via the peritoneal catheter port. EPIC was only offered to patients with PMP. No EPIC was offered to patients with other types of malignancies. For PMP, 5-fluorouracil 650 mg/m^2^ IP combined with 50 mEq sodium bicarbonate was administered from days 2 to 6. A total of five cycles were given. Normally, EPIC was administered either in the ICU or high dependency unit (HDU). The intraperitoneal chemotherapy was allowed to dwell for 23 h before it was removed by closed suction drains over the course of 1 h. The next installation was commenced once the abdomen was cleared of fluid as completely as possible.

### Statistical analysis

All statistical analyses were performed using IBM SPSS for Windows version 22. Comparison of normally distributed variables was performed using analysis of variance (one-way ANOVA) test. Categorical variables were analysed using the chi-square test or Fisher’s exact test where appropriate. Perioperative morbidity and mortality were the primary outcomes of this study. Hospital mortality was defined as any death that occurred during the same hospital admission for CRS. Median survival was calculated based on the date of death or last follow-up in the unit of months. Survival analysis was performed using the Kaplan-Meier curves and log-rank test for comparison. A significant difference was defined as *p* value less than 0.05.

## Results

### Descriptive characteristics

Six hundred eleven patients underwent CRS combined with PIC. The mean age of the study group was 52.8 years old (standard deviation (SD) = 13.1, median = 54.0, range = 71). There were 262 males (42.9 %) and 349 females (57.1 %). The diagnoses included CRC (*n* = 184, 30.1 %), PMP (*n* = 157, 25.7 %), mesothelioma (*n* = 53, 8.7 %), appendiceal cancer (*n* = 137, 22.4 %) and other cancers (*n* = 80, 13.1 %).

Patients’ background characteristics were summarised in Table 1. In the elderly group, the mean PCI was 16.5 (SD = 11.7) and a complete cytoreduction (CC0) was achieved in 77 % patients. The younger group had a mean PCI of 18.4 (SD = 11.2), and a CC0 was achieved in 81 % of young patients. There was no significant statistical difference in terms of year of operation, diagnosis, mean PCI and CC score between the two groups (*p = 0.235*, *0.118*, *0.095* and *0.264*, respectively) (Table 1). However, patients who were 65 years of age or older required significantly lower mean transfusion units (4.8, SD = 6.5) as compared with patients aged less than 65 years old (6.3, SD = 7.8) (*p = 0.048*).

**Table 1:** Background characteristics of patients in group 1 and group 2

	Group 1 (< 65 years old)	Group 2 (≥ 65 years old)	*p*
*Total n = 611*			
Number *n* (%)	487 (79.7)	124 (20.3)	
Gender *n* (%)			0.028
Male	198 (40.7)	64 (51.6)	
Female	289 (59.3)	60 (48.4)	
Age mean (range)	48.4 (14–64)	69.9 (65–85)	
Year of operation *n* (%)			0.235
1996–2002	30 (6.2)	3 (2.4)	
2003–2007	126 (25.9)	31 (25.0)	
2008–2013	331 (68.0)	90 (72.6)	
Diagnosis *n* (%)			0.118
CRC (*n* = 184)	137 (28.1)	47 (37.9)	
PMP (*n* = 157)	124 (25.5)	33 (26.6)	
Mesothelioma (*n* = 53)	47 (9.7)	6 (4.8)	
Appendiceal cancer (*n* = 137)	111 (22.8)	26 (21.0)	
Others (*n* = 80)	68 (14.0)	12 (9.7)	
HIPEC *n* (%)	424 (87.1)	95 (76.6)	0.004
EPIC *n* (%)	237 (48.8)	60 (48.4)	
PCI mean (SD)	18.4 (11.2)	16.5 (1.7)	0.095
CC *n* (%)			0.264
0	394 (81.7)	95 (77.2)	
1	75 (15.6)	22 (17.9)	
2	9 (1.9)	6 (4.9)	
3	4 (0.8)	0 (0)	
Transfusion mean (SD)	6.3 (7.8)	4.8 (6.5)	0.048

### Perioperative mortality results

Table 2 demonstrates the morbidity and mortality rates. The total hospital mortality was 2.3 % (*n* = 14). The hospital mortality rate in the patients aged older than 70 years old was 3 %, which is higher than that of the other younger patients. Nevertheless, such a difference in terms of mortality between the groups did not reach a statistical significance (*p = 0.607*).

**Table 2:** Mortality and morbidity results of patients in group 1 and group 2

	Group 1 (< 65 years old)	Group 2 (≥ 65 years old)	*p*
Total hospital stay (days) median (range)	22.0 (5–306)	22.5 (6–157)	0.925
ICU stay (days) median (range)	2.0 (0–71)	2.0 (1–101)	0.629
HDU stay (days) median (range)	3.0 (0–39)	2.0 (0–39)	0.831
Hospital death *n* (%)	10 (2)	4 (3)	0.607
Complication grade *n* (%)			0.644
0	88 (18)	25 (20)	
I	19 (4)	8 (6)	
II	166 (34)	42 (34)	
III	125 (26)	31 (25)	
IV	89 (18)	18 (15)	
Tumour-related OS median (months) (95 % CI)			0.889
CRC (*n* = 184)	27.0 (20.4–33.6)	42.0 (30.1–53.3)	0.130
PMP (*n* = 157)	MNR	MNR	0.356
Mesothelioma (*n* = 53)	62.0 (17.2–106.8)	22.0 (5.5–38.5)	0.186
Appendiceal cancer (*n* = 137)	64.0 (55.4–72.6)	43.0 (28.5–57.5)	0.475
Others (*n* = 80)	25.0 (16.8–33.2)	24.0 (4.8–43.2)	0.664

### Perioperative morbidity results

The mean of total hospital stay was 29 days (SD = 26, median = 22, range = 301). There was no significant difference among the three groups in terms of total hospital stay, ICU stay and HDU stay (*p = 0.607*, *0.629* and *0.831*, respectively) (Table 2).

Twenty percent of the elderly patients did not experience any complication, whereas only 18 % of patients in the younger group experienced no complications. The most common complications included intra-abdominal collection (*n* = 230, 37.6 %), infection (*n* = 209, 34.2 %), pleural effusion (*n* = 171, 28.0 %), fistula formation (*n* = 66, 10.8 %) and ileus (*n* = 69, 11.3 %). There was no significant difference in terms of morbidity rates between the two groups. (*p = 0.644*) (Table 2).

In terms of subgroup analysis, Table 3 summaries the background characteristics and clinical outcomes of the patients who were 75 years of age or older at the time of surgery. The morbidity results of our subgroup were similar to the results of patients who were less than 65 years old. However, the hospital mortality in the subgroup was higher (5 % *n* = 1) than that of those who were less than 65 years old (2 % *n* = 10) (Table 3).

**Table 3:** Subgroup analysis: background characteristics and clinical outcomes of patients who are 75 years of age or older at the time of surgery

Age ≥75	
*N* = 20	
Gender *n* (%)	
Male	12 (60.0)
Female	8 (40.0)
Age mean (range, SD)	76.7 (75–85)
Year of operation *n* (%)	
1996–2002	0
2003–2007	6 (30.0)
2008–2013	14 (70.0)
Diagnosis *n* (%)	
CRC (*n* = 184)	5 (25.0)
PMP (*n* = 157)	6 (30.0)
Mesothelioma (*n* = 53)	2 (10.0)
Appendiceal cancer (*n* = 137)	6 (30.0)
Others (*n* = 80)	1 (5.0)
HIPEC *n* (%)	9 (45.0)
EPIC *n* (%)	5 (25.0)
PCI mean (SD)	22.8 (13.0)
CC *n* (%)	
0	11 (55.0)
1	5 (25.0)
2	3 (15.0)
3	0 (0)
Transfusion mean (SD)	5.2 (6.2)
Total hospital stay (days) median (range)	20.0 (9–157)
ICU stay (days) median (range)	2.0 (1–101)
HDU stay (days) median (range)	1.0 (0–20)
Hospital death *n* (%)	1 (5)
Complication grade *n* (%)	
0	5 (25.0)
I	1 (5.0)
II	4 (20.0)
III	6 (30.0)
IV	4 (20.0)
Tumour-related OS median (months) (95 %)	35.0 (17.3–52.7)
5-year OS (%)	18.6

### Survival

Survival results were summarised in Table 2. Median overall survival (OS) of patients who were less than 65 years old was 58.0 months (95 %CI = 47.0–68.9) with a 5-year OS of 47.7 % whereas the elderly group had a median OS of 43 months (95 %CI=38.1–47.9) with a 5-year OS of 42.9 %. However, such a difference did not reach a statistical significance (*p = 0.698*). Cox regression analysis showed that age alone is not a prognostic factor for survival of peritoneal carcinomatosis (*p = 0.795*).

**Fig. 1:**
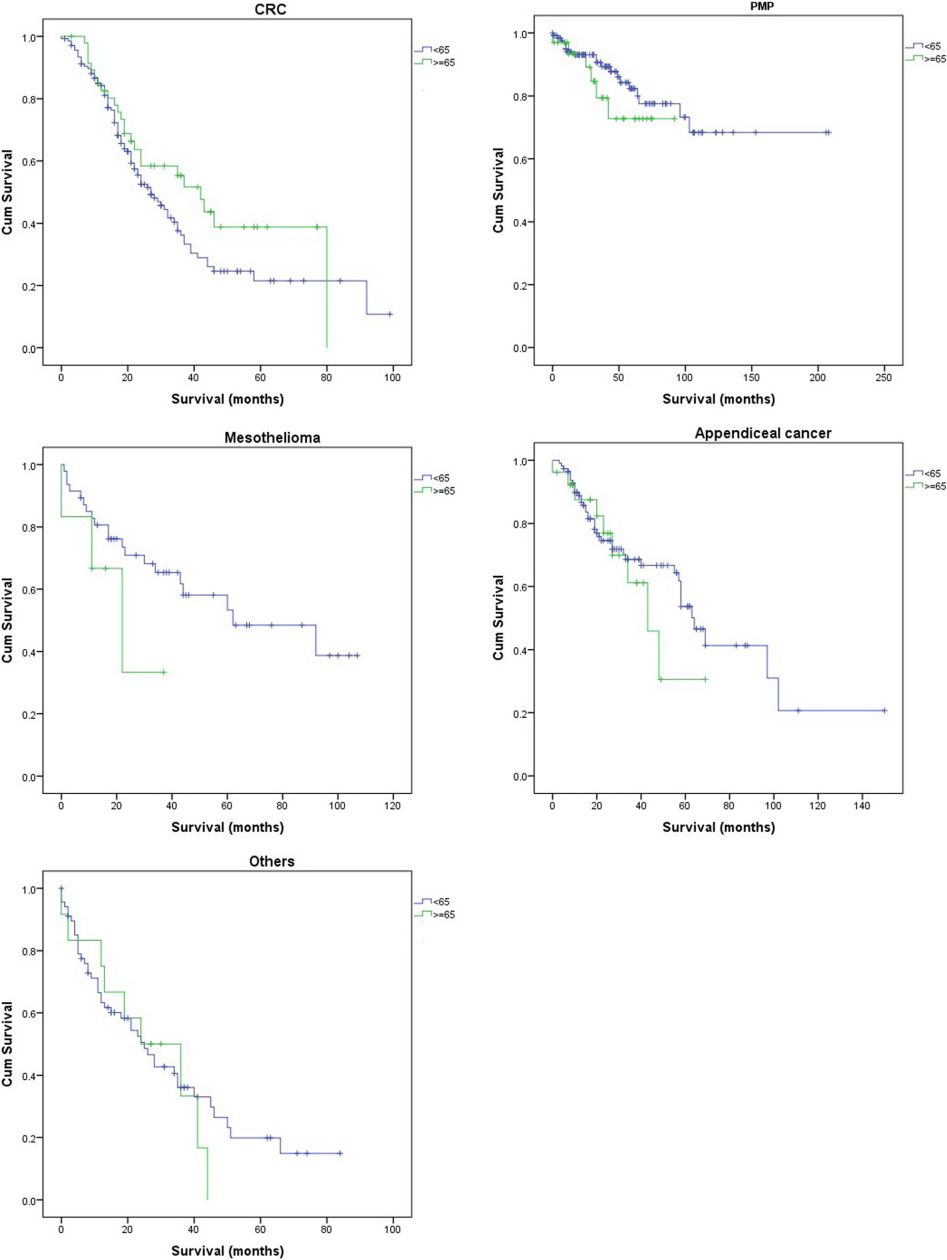
Survival curves for two age groups

The 5-year OS for patients with CRC was shown to be 26.8 %. The 10-year survival rate for patients with PMP was 67.4 %. The overall 5-year OS for this group of patients was 50.9 %. The overall 5-year OS for patients with appendiceal cancer was 50.5 %. The overall 5-year OS for patients with other types of cancers was 16.8 % (Fig. 1).

In terms of subgroup analysis, the patients who were 75 years of age or older had a median OS of 35.0 months (95 %CI = 17.3–52.7) with a 5-year OS of 18.6 %. Further survival analysis based on tumour types was not performed due to the limited number patients in each diagnosis group.

## Discussion

The Australian population is growing and ageing rapidly, requiring more and more healthcare resources. The health cost is expected to be doubled by 2051 as compared to costs in 1996 [13]. Cancer-related surgery is expected to rise substantially in the next few decades. According to the Australian Bureau of Statistics, life expectancy (additional years of life) of a male and a female reaching 65 is 18.7 and 21.8, respectively [14]. In the past, age alone has been considered as a limiting factor for major abdominal surgery. One previous study has shown that elderly patients (those older than 70 years old) were at higher risk of postoperative complications after non-cardiac surgery [15]. Given the fact that CRS is performed to remove all macroscopic disease, there are usually extensive intra-abdominal areas involved. Morbidity and mortality of CRC and PIC is not insignificant [16]. Thus, shorter survival would have been expected in the elderly patients. However, our study did not find a significant survival difference between the elderly patients and the younger patients.

Our mortality rate for the elderly patients was similar to the rate shown in the systemic review done by Chua et al. [6]. They reviewed the morbidity and mortality outcomes of CRS and HIPEC from all institutions and showed the mortality rate ranged from 0.9–5.8 % in the tertiary centres. Mortality of the elderly patients in our study was 3 %, which was not statistically different from the mortality of the younger patients after undergoing CRS and PIC. Similar mortality and morbidity rates for performing CRS and PIC for elderly patients as compared to younger patients have also been reported previously [7–9, 16, 17]. Although the mortality of our patients in the subgroup (i.e. ≥75 years old) is higher than our overall mortality, it is still consistent with the range of mortality reported by Chua et al. [6]. Despite the differences in mortality, the morbidity results of our subgroup are similar to the results of patients who were less than 65 years old. In terms of survival, the median OS and 5-year OS of the patients in the subgroup are shorter than that of patients who were 65 years of age or older. However, it is worth noting that the mean PCI in our subgroup was much higher than that of patients who were less than 65 years old or those who were 65 years of age or older. The lower rate of complete cytoreduction and higher PCI may have contributed to the higher hospital mortality and the lower 5-year OS in patients who were 75 years of age or older. Also, the 5-year OS may not reflect the true survival of these patients in our subgroup due to the fact that different cancer types could have contributed differently to the OS. Unfortunately, we could not perform survival analysis using tumour types in our subgroup due to the limited number of patients in each diagnosis group.

Several previous studies have assessed the safety of CRS and HIPEC for the elderly patients. The most recent study was done by Spiliotis et al., in which they analysed the results of 100 patients and 30 of which were over 70 years of age [7]. They suggested that it may be safe to perform CRC and HIPEC for the elderly population with a careful selection. An Italian study done by Macri et al. presented a retrospective analysis of 30 patients who underwent CRS and HIPEC. They suggested that a combination of CRS and HIPEC is suitable for the elderly population by comparing patients aged less than 65 years old against 11 patients aged older than 65 years old [9]. Also, Muller et al. [8] did a retrospective analysis of 44 patients over 65 and suggested that age alone should not preclude patients from cytoreductive surgery. However, they did not compare their results against those who are 65 years of age or younger. Although our findings are consistent with these studies, it should be noted that suitability of our patients for CRS and PIC was discussed during weekly peritonectomy meetings. Only selected patients were offered this combined therapy at our centre. Similar mortality, morbidity and survival outcomes between our age groups may have reflected the careful selection process. Thus, our findings suggest that CRS combined with PIC can be safely performed in the patients who are 65 years of age or above with an acceptable mortality and morbidity after a careful patient selection.

To our knowledge, our study is the largest study of patients over 65 years old in the literature. However, we acknowledge that there were some limitations in this study. This was a retrospective study conducted at a single centre. There was undoubtedly selection bias during the study. Also, clearly there will be a referral bias, and we are not sure the number of elderly patients who were not referred to our centre and turned down due to their age alone. Moreover, our results were obtained in a centre with an experience of more than 900 patients. The learning curve and volume outcome effects in major surgery and potentially peritonectomy may be potentially important in this group of patients.

## Conclusions

The balance between surgery-associated risks and potential benefits is always paramount. Our study suggests that CRS and PIC can be safely performed for the elderly patients. However, the decision to offer this combined therapy is often complicated by other factors including co-existing morbidities. Thus, CRS and PIC should only be offered to a subset of elderly patients after careful selection and discussion during a multidisciplinary team meeting. In addition, variations in national funding for peritonectomy should also be considered since it further complicates the availability of CRS and PIC to suitable elderly patients. The healthcare cost of cytoreductive surgery and HIPEC is acknowledged (on average between AUD$44,668 and AUD$92,308 depending on the type of malignancy), and societal decisions need to be made on its provision [17]. However, our results indicate that in patients over 65 years old, good short- and long-term results are achieved. Hence, age alone should not be the single exclusion criterion but rather taken into consideration along with other factors to determine the suitability of elderly patients to undergo CRS and PIC.
